# Ubiquitin Proteasome System BTB-Adaptor Protein Insomniac Modifies Seizure Severity and Response to the Ketogenic Diet in a *Drosophila* Model of Mitochondrial Encephalomyopathy

**DOI:** 10.3390/genes17101185

**Published:** 2026-09-24

**Authors:** Keri J. Fogle, Brynn Urban, Dana M. Mumford, Michael J. Palladino

**Affiliations:** 1 Department of Pharmacology & Chemical Biology, School of Medicine, University of Pittsburgh, Pittsburgh, PA 15260, USA; kjf38@pitt.edu (K.J.F.); burban@butler.edu (B.U.); dmm241@pitt.edu (D.M.M.); 2 Pittsburgh Institute for Neurodegenerative Diseases, School of Medicine, University of Pittsburgh, Pittsburgh, PA 15260, USA

**Keywords:** ubiquitin proteasome system, mitochondrial encephalomyopathy, insomniac, Cullin-3, seizures, ketogenic diet, advanced glycation end products (AGEs), β-hydroxybutyrylation, epigenetics

## Abstract

**Background/Objectives:** The ubiquitin proteasome system (UPS) serves critical cellular homeostatic functions that contribute to neurological health, including neuronal metabolism, synaptic plasticity, and the molecular circadian clock. The UPS is tightly regulated to ensure the correct balance of healthy proteins and those targeted for degradation and recycling; malfunction in this quality control process can contribute to disease states such as neurodegeneration and cancer. Target specificity in this system is due in part to ubiquitin ligase adaptor proteins, such as those of the BTB superfamily of proteins, which connect substrates to the larger proteasomal complex. In *Drosophila*, the BTB family member insomniac (human orthologs KTCD2, 5, and 17) has been shown to regulate sleep, arousal, and synaptic plasticity, but has not been examined for a role in neuronal excitability disease phenotypes. **Methods:** We utilized a genetic approach to interrogate neurobehavioral dysfunction in mitochondrial encephalomyopathy (ME) and the ability of dietary therapy to modulate this dysfunction. **Results:** Here, we report that genetic interference with insomniac modifies seizure-like behavior in a neuronal-subtype-specific manner in the *ATP6^1^* model of ME, ameliorating seizure severity when knocked down in GABAergic or dopaminergic neurons. Furthermore, while *ATP6^1^* typically responds to ketogenic dietary therapy with a robust reduction in seizure phenotype, we report that knockdown of insomniac or its binding partner Cullin RING Ligase 3 (Cul3) in neurons ablates this effect entirely, a previously unreported role for the UPS in downstream neurophysiological effects of the KD. **Conclusions:** These results implicate UPS BTB adaptor proteins as therapeutic targets in seizure pathology and identify them as a novel contributor to the ketogenic suppression of neuronal hyperexcitability. These two neurological roles are likely to be proceeding via cellular and molecularly distinct pathways, including a non-CRL3-based mechanism of seizure modulation.

## 1. Introduction

The ubiquitin proteasome system (UPS) is a critical cellular system for regulating protein homeostasis, ensuring correct quantity and functionality. By post-translationally tagging unneeded, malfunctioning, or misfolded proteins with ubiquitin, this system executes targeted protein quality control via degradation and recycling. This system has important roles in fundamental neurological processes such as the synaptic plasticity underlying learning and memory and the protein-level cycling underlying circadian rhythms. Disruptions or decline in the proper functioning of the UPS is implicated in aging and its associated diseases, including cancer, cardiovascular disease, stroke, and neurodegeneration [1,2,3,4,5,6,7,8,9]. Specifically, underperformance of the UPS is hypothesized to play key roles in aggregation diseases such as Alzheimer’s, Parkinson’s, and Huntington’s Diseases, as well as amyotrophic lateral sclerosis and frontotemporal dementia, where an abnormal accumulation of proteins interferes with neuronal function [2,4,5,10,11,12,13]. Additionally, the UPS has emerged as a key player in some types of epilepsy, where protein turnover at overactive synapses may be compromised, with the proteasome unable to keep up with excess protein expression, leading to the hypothesis that UPS dysfunction may underlie some seizure activity [14]. Despite this, many specific adaptor proteins critical to the specificity and precision of the UPS have not been investigated in seizure pathophysiology.

The UPS is also a critical regulator of mitochondrial function. Mitochondria are susceptible to proteotoxic stress, in which aberrant proteins affect their ability to produce energy via oxidative phosphorylation and perform other critical tasks [15]. Unresolved, this stress can lead to mitochondrial disease or dysfunction that contributes to neurodegeneration [16]. While mitochondria have a non-UPS internal proteostasis system, they also rely on the UPS in several key ways. First, most mitochondrial proteins are encoded by the nuclear genome and translated in the cytosol before import. Second, mitochondria are dynamic structures that often undergo fission or fusion in response to cellular demands; the proteins that regulate these processes are susceptible to UPS quality control and regulation [17]. The mitochondria themselves can also be targeted for mitophagy, a controlled elimination process to remove damaged organelles that involves coordination between the UPS and lysosomal degradation [18,19,20,21,22]. Parkinson’s-associated genes *PINK1* and *Parkin* encode critical proteins that help coordinate this process, working together to tag damaged mitochondria and poly-ubiquitinate them, sealing their fate in the lysosome [23,24,25]. Importantly, the UPS is itself a highly energetically demanding system, making protein quality control and mitochondrial function interdependent processes in which damage to one may exacerbate disease states in the other.

Because of the critical function it provides and the impactful consequences of its errors, the UPS must be highly precise, efficiently targeting only those proteins that need to be degraded. This target specificity is accomplished by the families of adaptor proteins, which connect ubiquitin ligase complexes to only a few selected substrates. One such family is those with a protein–protein interaction site known as the BTB domain (Broad-Complex, Tramtrack, and Bric à brac), which is conserved across invertebrates and vertebrates, including both *Drosophila* and humans.

In *Drosophila*, the BTB family adaptor protein insomniac (inc) binds to Cullin-3 (Cul3), a core scaffolding protein for the Cullin RING Ligase 3 (CRL3) complex, at its N-terminus and to substrates targeted for degradation at the C-terminus [26]. The *insomniac* (*inc*) gene has three human orthologs (*KCTD2*, *KCTD5*, and *KCTD17*), which are expressed in the mammalian nervous system [27] and can rescue sleep defects in *Drosophila inc* mutants. These orthologs are associated with human diseases including myoclonus dystonia, Alzheimer’s disease risk, and abnormal sleeping patterns [28,29,30]. Cul3 is a well-established genetic marker in neurological and neurodevelopmental disease, including autism spectrum disorder, developmental delays, schizophrenia, and rare epileptic encephalomyopathies [31,32,33,34,35,36].

In flies, *inc* was so-named after it emerged from a forward genetic screen as a powerful modulator of sleep quantity and quality [37]; it also displays a reduced compensatory response to deprivation and decreased arousal threshold to stimuli [38]. While the exact cellular and molecular processes underlying inc’s profound sleep phenotypes remain to be fully elucidated, several plausible mechanisms arise from its known functions and expression patterns. For example, *inc* is expressed in the sleep homeostasis-critical central complex [27], PDF-expressing neurons, which are key circadian regulators of sleep and arousal [27,39,40], and dopaminergic arousal pathways [38]. Despite its crucial role in sleep, *inc* is not a component of the *Drosophila* molecular clock and has no known interactions with core clock proteins, nor is it a circadian-regulated gene [37].

Additionally, loss of inc is associated with neurodevelopmental defects. The inc protein is known to traffic to neuronal projections and synapses, and its loss leads to synapse deformation [27]. These developmental issues are particularly severe in the mushroom bodies, structures critical for sensory integration, learning and memory, and sleep [41]. Loss of inc during a critical developmental period results in anatomical defects: neurons are produced in excess leading to overgrowth and poor circuit assembly with a particular increase in cholinergic synapses [41,42]. Intriguingly, this overgrowth, while likely contributing to impaired sleep, also results in overperformance in associative learning and memory tasks [43]. The inc protein may also regulate synaptic function through mono-ubiquitination in addition to the poly-ubiquitination process of the UPS which targets proteins for degradation, as it has been shown to regulate retrograde signaling after glutamate receptor inactivation at the neuromuscular junction [44].

Inc also participates in the integration of sleep and metabolism, especially under conditions of stress and starvation. During starvation, sleep is suppressed to encourage foraging behavior, a behavior mediated by a single pair of Lateral Horn Leucokinin (LHLK) neurons; *inc* is downregulated in these neurons during starvation, and RNAi-based knockdown in only these two neurons is sufficient to reduce sleep [45]. Additionally, *inc* is present in corazonin (CRZ, pGlu-TFQYSRGWTNH2)-positive neurons, which are known to communicate with insulin-producing cells and regulate energy levels and response to starvation [46].

Given *inc*’s established roles in sleep, neurodevelopment, synaptic function, and response to bioenergetic stress, we included it in an RNAi-based genetic screen for modifiers of neurological phenotypes in the *ATP6^1^ Drosophila* model of mitochondrial encephalomyopathy. This model, which arises from a mutation in the *ATP6* gene of the mitochondrial chromosome which encodes for the proton-passing subunit of Complex V, exhibits severe, progressive, stress-induced seizure behavior [47,48,49]. Interestingly, *ATP6^1^* seizures are dramatically reduced when the animals are fed a ketogenic diet (KD) [50,51,52].

The metabolically based etiology of these seizures is still unclear, but may involve the altered modulation of metabolically sensitive ion channels such as K_ATP_, toxic buildup of intermediates such as lactic acid and methylglyoxal due to overreliance on glycolysis, bioenergetic stress at the synapse, neurotransmitter imbalance, or other mechanisms. The ketogenic diet forces cells to limit glycolysis and favor the catabolism of lipids, which may improve hyperexcitation phenotypes by bolstering anaplerosis or mitochondrial biogenesis, altering gene expression and post-translational modifications, increasing ketone body and lipid modulation of ion channels and neuronal receptors, and reducing the toxic byproducts of glycolysis [50,51,53,54,55,56,57,58,59,60,61,62,63,64,65,66]. The latter may lower carbonyl stress, reducing the production of advanced glycation end products (AGEs), which can covalently alter proteins, rendering them both non-functional and resistant to UPS degradation [67,68,69,70,71,72,73].

In a genetic screen of several hundred genes, *inc* emerged as an intriguing positive hit, modifying basal seizure severity and the response to the ketogenic diet. These results were validated with a global classical allele of *inc* (*inc^1^*). However, the mechanistic underpinnings of these two phenotypes remained unknown; therefore, in the current study, we addressed two fundamental mechanistic questions: whether *inc*’s effects were occurring via its association with CRL3 (and thus the UPS); and whether specific neuronal subpopulations could be identified as key mediators of these neurological results, thus providing targets for further investigation. We hypothesized that genetic interference with *Cullin-3* would replicate *inc* knockdown/out given their known molecular cooperation in targeting proteins for degradation, and that *inc*’s effects on seizures would be significant in neuronal populations responsible for excitability, arousal, and seizure genesis.

We found that pan-neuronal knockdown of *inc* significantly worsened *ATP6^1^* seizures, but was not replicated by the pan-neuronal RNAi knockdown of inc-binding partner in the CRL complex, Cul3, suggesting a previously unidentified molecular role for inc. Additionally, *inc* emerged as a robust inhibitor of the therapeutic effect of the ketogenic diet on seizure severity and, in contrast to the basal effect on seizures, this was replicated by Cul3 knockdown, potentially indicating separate mechanisms for the inc modulation of the basal vs. therapeutic phenotypes. A screen of neuronal subpopulations revealed that while knockdown in the mushroom bodies and cholinergic neurons worsened seizures, intriguingly, knockdown in either GABAergic or dopaminergic neurons alone ameliorated them without preserving the ketogenic diet response. Overall, these results reveal a novel role for inc in the seizures of mitochondrial disease and suggest, pending further mechanistic investigation, that it could be a therapeutic target, especially in GABAergic and dopaminergic neurons. Furthermore, these results may implicate the ubiquitin proteasome system as contributing to the dynamic and profound downstream metabolic changes that result from dietary-induced ketogenesis.

## 2. Materials and Methods

### 2.1. Drosophila Media, Maintenance, and Genetics

Strains, matings, and experimental flies were maintained in standard media comprising agar, corn meal, dextrose, sucrose, dry yeast, and molasses, with propionic acid, phosphoric acid, and Tegosept (Genesee Scientific Corporation, El Cajon, CA, USA) added as fungicides. The Tegosept (methylparaben) concentration utilized (0.15% *w*/*v*) is standard in *Drosophila* media and below examined thresholds for toxicity and developmental effects [74,75,76].

Flies are maintained in a light and temperature-controlled room under a 12:12LD schedule. In all reported genotypes, “*ATP6^1^*” or “*ATP6[1]*” refers to the mitochondrially encoded gene mutation in *ATP6* and is denoted first, separated from the nuclear chromosomes by standard semi-colon notation. The *ATP6^1^* strain used in all experiments was a mitochondrial recombinant generated by the O’Farrell lab and described previously [77,78].

The control strain is *ATP6*^+^; *w^1118^*. Female *ATP6^1^*; w*; *GAL4* driver lines were mated with males of this line to generate “driver control” lines. *ElavGAL4* (BL#8765) GADGAL4 (BL#51630), ChATGAL4 (BL#56500), THGAL4 (BL#8848), KenyonGAL4 (BL#50742) and *UAS-RNAi* lines for *inc* (BL#57173) and *Cullin-3* (BL#36684) were obtained from Bloomington FlyStocks (Bloomington, IN, USA) and are in the Valium20 vector. The inc classical allele *(inc^1^*) is an amorphic allele [37,38], also obtained from Bloomington (BL#18307). The *UAS-inc* overexpression line was generated by FlyORF (University of Zurich). *UAS*/*GAL4* transgenic progeny were generated using standard genetic methods with *ATP6^1^*; w*; *GAL4* stable line virgin females mated to males with the *UAS* construct of interest. A total of 5–6 equivalent crosses were set up to generate the experimental progeny.

Administration of the ketogenic diet was performed as described previously [50,51,52]. Briefly, the ketogenic diet is administered in a standard vial with a 1.2% agar plug with 0.1% Tegosept for hydration and mold inhibition. Nutrients are supplied in a 3:1 lipid:protein + carbohydrates paste, provided in a 1 cm plastic cup (generated by clipping a 1 mL disposable filter pipette tip), with a recipe as follows: 75% unrefined coconut oil, 13.75% corn meal, 7.5% nutritional yeast, 3.75% sucrose (all percentages are in *w*/*w*). All groups of flies represented in this data set were given the same access to the same volume of ketogenic paste *ad libitum* and in excess of what they could consume in three days. Ingredients were obtained from Fisher Scientific (Waltham, MA, USA). Flies were maintained on this ketogenic media from day 9 and through testing on Days 15–21.

### 2.2. Mechanical Stress Seizure Induction

Male and female flies are collected at eclosion (day 0) and kept in groups of 20–50 on standard media until they reach age 9 d when they are separated at random into groups of 5–8 mixed-sex individuals and placed in these small groups onto either regular media or ketogenic media. Flies are excluded at this stage if they are dead or visibly injured. Flies are maintained under these conditions with new media every 3 days for the duration of the experiment. At room temperature, the bang-sensitive phenotype begins to emerge at Day 12, is accurately measurable by Day 15 (early disease), and worsens at Day 18 (intermediate disease) and Day 21 (advanced disease). Phenotype progression and severity depends on disease model and temperature (room temp 21–22 °C or 69–72 °F or an incubator at 25 °C or 77 °F, controlled within a given experiment). If the flies are kept at 25 °C, this timeline is accelerated by approximately 3 days.

Stress sensitivity or bang sensitivity (BS) experiments to assay stress-induced seizures were conducted as originally described by Ganetzky and Wu [79], with some modifications that have been described in detail previously [47,50]. Briefly, flies are placed in empty plastic, cotton-plugged vials in groups of 5–8, then vortexed at maximum speed for 20 s on a Vortex Genie 2 (Scientific Industries, Inc. Bohemia, NY, USA). Bang-sensitive flies exhibit paralysis, convulsions, or a combination of the two, while wild type flies recover immediately following the stimulus. *ATP6^1^* and derived lines exhibit progressively longer periods of paralysis and convulsions; due to this phenotype, their seizure severity is measured by the parameter time to recovery (TTR), which measures how long it takes each fly to resume purposeful movements after paralysis and convulsion. Each fly’s recovery time is recorded and represented as one data point in the figures and the total number of flies is reported as *n* values in the figure legends. Phenotype progression and severity depends on disease model and temperature (room temp 21–22 °C or 69–72 °F or an incubator at 25 °C or 77 °F, controlled within a given experiment).

### 2.3. Data Analysis

Data are presented as bars with error bars representing mean ± standard error, with all individual data points contributing to the set overlaid. Data were tested for normality using the Shapiro–Wilk test and, as is typical for bang sensitivity data sets, the data failed normality testing. Therefore, multiple value comparisons were performed by Kruskal–Wallis tests followed by Dunn’s multiple comparisons tests for controls RD vs. KD, control RD vs. genetic manipulation RD, and genetic manipulation RD vs. KD. Data compilation, figure generation, and statistical tests were performed using Prism.

## 3. Results

### 3.1. Pan-Neuronal or Global Loss of Inc Leads to Worsened Seizure Phenotypes and Loss of Ketogenic Diet Response

To investigate (1) the etiology of seizures in mitochondrial encephalomyopathies, and (2) the cellular and molecular mechanisms underlying the therapeutic benefit of the ketogenic diet thereof, we undertook a reverse genetic screen by pan-neuronally driving RNAi against dozens of target channels, receptors, and other small molecules genes that may modify excitability and synaptic function in the *ATP6^1^* model of ME. We tested the seizure phenotype, as well as the ability of the ketogenic diet to reduce seizure severity compared to regular carbohydrate-based diet (RD), at a moderately advanced disease time point in the RNAi-knocked-down animals compared to the aged-matched driver-only controls, both in the *ATP6^1^* genetic background. The initial screen identified the *inc* gene as both a seizure and ketogenic diet modifier, worsening time to recovery under both RD and KD conditions compared to genetic controls.

To validate this result, we tested the seizure phenotype in flies fed either a regular carbohydrate-based diet (RD) or a coconut oil-based ketogenic diet (KD) over the full time course of the progressive mitochondrial disease in flies with pan-neuronally knocked down *inc* vs. driver-only controls. At all tested time points, in *ATP6^1^* driver control animals, the ketogenic diet (KD, blue dots) significantly reduced time to recovery from seizure compared to animals fed the regular diet (RD, black dots), (*p* = 0.0003 at Day 15, *p* = 0.0021 at Day 18, and *p* = 0.0005 at Day 21) (Figure 1A). In contrast, when *inc* was genetically knocked down in the brain, the KD (green dots) failed to reduce seizure time to recovery at Day 15 (*p* = 0.4336) and Day 18 (*p* = 0.1068), only showing therapeutic benefit in late disease vs. RD (red dots), Day 21 (*p* < 0.0001) (Figure 1A). Additionally, the initial observation of worsening of basal seizure phenotype was verified, with significantly lengthier seizures observed at Day 18 (*p* = 0.0171) and Day 21 (*p* < 0.0001) with knocked down *inc* vs. driver controls on regular media (black vs. red dots) (Figure 1A).

As a genetic validation of inc knockdown’s effects on seizures, we generated double-mutant animals with both the *ATP6^1^* mutation on the mitochondrial chromosome and the *inc^1^* global knockout allele. We performed the same time course experiment described above and found that in *ATP6^1^*; *inc*^+^ control flies the ketogenic diet significantly reduced seizure severity at all time points (*p* < 0.0001) (Figure 1B). In contrast, in *ATP6^1^*; *inc^1^* mutants, the KD failed to reduce seizures (*p* = 0.9999 at days 15 and 18, *p* = 0.8676 at Day 21, vs. regular media). Baseline RD seizures were also worsened in the *ATP6^1^*; *inc^1^* animals reaching significance at Day 15 (*p* = 0.0419) and Day 18 (*p* = 0.0393) (Figure 1B). Together, these results validate our initial finding that *inc* is a modulator of seizure severity and the ketogenic diet therapeutic response.

### 3.2. Neuronal Knockdown of Cul3 Replicates the Loss of KD Response

One fundamental question is whether the inc effects are occurring via a Cullin-3 mediated mechanism, inc’s known RING Ligase complex binding partner, which could implicate the UPS in both mitochondrial encephalomyopathy seizures and critical metabolism-excitability connections underlying the ketogenic diet. To address this, we performed seizure testing on pan-neuronally driven UAS-RNAi against *Cul3* animals fed regular and KD media. While *ATP6^1^*; *elav*/+ flies responded as expected to the KD (*p* = 0.0008 and 0.0044 vs. RD—black vs. blue dots—at Days 18 and 21, respectively), flies with knocked down Cul3 performed ***worse*** on the KD at Day 18 (*p* = 0.0196 vs. RD, green vs. red dots) and exhibited no change vs. RD at Day 21 (*p* > 0.9999) (Figure 2). This finding demonstrates that Cul3 and inc may be working in the same mechanistic pathway. In contrast, RNAi knockdown of *Cul3* did not mimic the effect of pan-neuronal inc loss on baseline seizure severity on RD. Comparison between driver controls and UAS-Cul3 RNAi flies yielded *p* values >0.9999 at all tested time points, indicating that loss of Cul3 in the brain does not worsen seizures in *ATP6^1^* flies (Figure 2).

### 3.3. Differential Effects of Inc Knockdown and Overexpression in Neuronal Subpopulations

To address the complexity of these results and generate targets for further mechanistic investigation, we restricted knockdown of *inc* to cholinergic, GABAergic, dopaminergic, or mushroom body neurons and tested seizure severity on RD and KD over the course of mitochondrial encephalomyopathy. These subpopulations were chosen for their known roles in excitation–inhibition balance and/or an established physiological role for inc within that population. For example, cholinergic neurons are the primary excitatory drivers in the *Drosophila* nervous system; furthermore, *inc* loss is known to cause synaptic deformation in this population. In *ATP6^1^*, knockdown of *inc* in cholinergic neurons worsened seizures for flies on RD at all time points compared to driver-only controls (*p* < 0.0001 Day 15; *p* = 0.0292 Day 18; *p* = 0.0017 Day 21, black vs. red dots) (Figure 3A). Effects of cholinergic *inc* loss on the response to the KD were mixed: On Day 15, the KD did reduce seizures, though the effect only barely reached significance (*p* = 0.0458 compared to RD for the same genotype, red vs. green dots) (Figure 3A). It is noteworthy that age-matched (Day 15) driver control populations did not achieve a significant reduction in response to the KD, though this could be due to a prohibitively mild phenotype. At Day 18, cholinergic *inc* loss results in a lack of KD response (*p* = 0.0796), while advanced disease Day 21 flies did respond (*p* = 0.0108), resulting, overall, in a less significant KD response (Figure 3A).

The mushroom bodies (MB), while primarily comprising cholinergic Kenyon cells, are a specialized structure important for sensory integration and seizures, as well as complex physiological phenomena such as sleep and memory formation. *Inc* loss has been shown to cause developmental issues in these structures, such as neuronal overgrowth, and behavioral changes such as altered memory performance. In our mitochondrial encephalomyopathy model, loss of MB inc resulted in a profound loss of KD response (*p* > 0.9999 at all time points vs. RD, green vs. red), while driver control animals displayed the expected robust response at Days 18 and 21 (*p* = 0.0087 and 0.0061, respectively) (Figure 3B). However, the effect on seizures on RD vs. controls follows a complex time course, with *inc* loss causing increased TTR at Day 15 (*p* = 0.0061), reduced TTR at Day 18 (*p* = 0.0019), and a strong trend toward worsening again at Day 21, though this effect does not reach significance (*p* = 0.1178) (Figure 3B). This complexity could reflect a multifaceted effect of inc knockdown on these critical structures (see Section 4).

In *Drosophila*, dopamine is an important determinant of sleep vs. wakefulness, and overall level of arousal in response to stimulus. Since inc mutants have behavioral defects in both of these phenotypes (reduced sleep and increased arousal), we asked whether dopaminergic populations contributed to the seizure and dietary response phenotypes in the genetic loss of inc. Intriguingly, in contrast to the effects observed in cholinergic knockdown, dopaminergic *inc* RNAi resulted in a reduction in seizure severity at Day 18 and Day 21 vs. RD driver controls (*p* < 0.0001 in both cases; *p* > 0.9999 for Day 15) (Figure 3C). Treatment with the KD did not elicit any further improvements in a dopaminergic knockdown vs. RD at any time point (*p* = 0.1434, 0.9999, and 0.1572 for Days 15, 18, and 21, respectively) (Figure 3C).

RNAi knockdown of *inc* in GABAergic neurons, the primary source of neuronal inhibition in both flies and vertebrates, also reduced baseline seizure severity compared to driver-only controls, though the effect was limited to Day 18 (*p* = 0.0005) (Figure 3D). Additionally, the KD was ineffective in these knockdowns, regardless of whether RD seizure severity was altered compared to controls (*p* = 0.9999 at days 15 and 18, *p* = 0.1584 at Day 21).

The ability of genetic knockdown of *inc* to suppress the seizure phenotype in *ATP6^1^* animals raises the intriguing possibility that this protein could be a therapeutic target, especially in GABAergic and dopaminergic neuronal populations. To investigate how malleable the effect of *inc* levels on seizure phenotypes may be, we used genetic overexpression to increase the amount of available *inc* protein in cholinergic, GABAergic, and dopaminergic neurons. Driving overexpression of *inc* in cholinergic neurons resulted in a worsened seizure phenotype (*p* = 0.0436 vs. driver controls at Day 15 and *p* = 0.0035 at Day 18) and loss of response to the KD (*p* = 0.3710, 0.9317, and 0.9999) (Figure 4A), a result that is qualitatively the same as RNAi knockdown in the same neurons (see Figure 3A). In contrast, overexpression of inc in GABAergic and dopaminergic neurons yielded effects that differed from the corresponding knockdown. In GABAergic neurons where knockdown improved seizure severity, overexpression worsened it at Day 15 (*p* = 0.0212 vs. driver controls) and at Day 21 (*p* = 0.0486) (Figure 4B). Interestingly, and in contrast to the effect in the genetic knockdowns (see Figure 3D), the KD amelioration evident as usual in driver controls (black vs. blue dots) is weakened but not completely ablated in the genetically overexpressed animals, showing a significant benefit at Day 21 (*p* = 0.0313) (Figure 4B). In dopaminergic neuron overexpression animals, seizure phenotype is neither repressed nor exacerbated (*p* = 0.3299, 0.5847, and 0.1438 vs. driver controls at Days 15, 18, and 21, respectively), while the ketogenic diet benefit, like in the RNAi knockdown conditions, is absent (*p* = 0.1397, 0.1057, 0.8094 vs. RD overexpression controls) (Figure 4C).

In summary, pan-neuronal or global genetic knockdown of inc worsens seizures and the response to the ketogenic diet; knockdown of Cul-3 replicates the KD effect but does not worsen the seizure baseline. In neuronal subpopulations, knockdown and overexpression (Table 1) do not necessarily produce complementary effects at the behavioral level, while all tested genetic manipulations of inc resulted in a worsened response to the KD.

## 4. Discussion

The ubiquitin proteasome system is a critical component of cellular homeostasis whose dysfunction is associated with aging, cancer, neurodegeneration, and diseases of manifold body systems. The Cullin RING Ligase 3 system adaptor Cullin-3 is specifically implicated in neurodevelopmental and neuronal excitation imbalance disorders such as autism, schizophrenia, and epilepsy. In *Drosophila*, its substrate-binding partner inc is associated with compromised sleep, synaptic overgrowth, and altered response to bioenergetic stress. Here, we identify two previously undescribed roles for this protein: (1) a modulator of seizures associated with mitochondrial disease; and (2) a facilitator of the amelioration of these seizures by the ketogenic diet. Intriguingly, only the effect on the ketogenic diet is replicated by the genetic knockdown of Cul-3, suggesting a non-CRL3 mechanism for seizure modification.

### 4.1. Inc Loss in Cholinergic and Mushroom Body Neurons Worsens Seizures

When knocked down pan-neuronally using the *elavGAL4* driver or knocked out globally by the classical allele *inc^1^*, loss of inc in the *ATP6^1^* animals worsens seizures and ablates the previously established, robust, therapeutic improvement imbued by the switch to a high-fat, low carbohydrate media (ketogenic diet). However, when *inc* knockdown is restricted to specific neuronal subpopulations, a more complex picture of its effects emerges.

Knockdown in cholinergic neurons led to a dramatic worsening of seizure phenotype across disease progression, a result that is cohesive with previous reports that its loss induced cholinergic synaptic deformation and overgrowth [41,42,43]. One plausible, though so far untested, explanation is that, as the primary excitatory neurotransmitter in the *Drosophila* brain, its excess or otherwise abnormal activity could exacerbate the imbalance in excitation–inhibition in the mitochondrial encephalomyopathy model.

The Kenyon cells are a subset of these cholinergic neurons that comprise the mushroom bodies, central brain areas critical for sensory integration, memory, and sleep that exhibit neurodevelopmental issues upon loss of inc during an early developmental window. Strikingly, while this altered development likely contributes to sleep loss and other neurological issues, *inc* mutants exhibit improved learning and memory performance [43]. Our Kenyon-cell targeted knockdown of *inc* led to a complex time course of effects on stimulation-induced seizures in *ATP6^1^* animals, with significant seizure reduction at disease onset that was replaced instead with increased severity in intermediate and advanced disease. Possible explanations include that the bidirectional nature of this effect may reflect inc’s interaction with the changing metabolic landscape as mitochondrial encephalomyopathy worsens with time, or dynamic shifts in the contribution of the mushroom bodies to pathophysiology. It is possible, for example, that effects of sleep loss or excess sensory integration become more prominent in advanced disease.

### 4.2. Inc Loss in GABAergic and Dopaminergic Populations Improves Seizure Phenotypes

Intriguingly, knockdown of *inc* in two neuronal subpopulations—GABAergic neurons and dopaminergic neurons—reduced seizure severity during disease progression. Given that loss of inc leads to neuronal overgrowth in the mushroom bodies, one plausible hypothesis for this effect may be an analogous increase in GABAergic neuron availability, reducing the excitation that leads to seizures. While no evidence of this overgrowth was found in one study [42], it remains a possibility that subtle, location-restricted, or mitochondrially related effects on GABAergic populations occur in our model. Of note, in cultured mouse hippocampal neurons, genetic loss of *Cul3* led to a decreased volume of GABAergic synapses, indicating that these can be dynamically regulated by the Cul3 complex [36].

In *Drosophila*, dopaminergic signaling to both the fan-shaped body and the mushroom bodies regulate sleep and wakefulness. Signaling to the fan-shaped body increases arousal levels and promotes wakefulness by the DopR1-mediated hyperpolarization of sleep promoting neurons [80,81], while inputs to the mushroom bodies can delay or suppress sleep in response to social, sensory, or metabolic cues. In *inc* mutants, sleep defects are not worsened by a null mutation in the transporter that clears the neurotransmitter from the cleft (DATfmn) [37], indicating they may be part of the same pathway, while inhibition of dopamine synthesis by tyrosine hydroxylase inhibitors can suppress inc’s sleep phenotypes [38]. Mutants of *inc* also display a reduced threshold to arousal from sleep by a mechanical stimulus [38]. Therefore, inc is likely to regulate dopaminergic activity as it pertains to sleep and arousal; here, we found that the RNAi knockdown of *inc* in dopaminergic neurons also suppressed seizure severity. Further work will be necessary to examine subpopulations of dopaminergic neurons to determine whether this effect is occurring via sleep or arousal signaling to the central complex, the mushroom bodies, the circadian circuit, or other, non-sleep related pathways. For example, *Cul3*-deficient mice exhibit enhanced dopaminergic signaling and elevated excitability in ventral tegmental neurons [33]. While dopamine is not generally regarded as a major player in seizures or epileptogenesis, its modulatory role has been well-noted [82,83], especially in seizures originating in the limbic system [84]. Furthermore, anti-convulsant properties of dopamine-elevating drugs such as amphetamines, L-DOPA, and anti-Parkinson’s drugs is well-established [84], and dopamine system viability as an anti-epileptic drug target is under active investigation [85].

Overall, *inc* knockdown’s complex, subtype-specific effects on seizures provides evidence that the protein is a critical contributor to excitation–inhibition balance, at least in the context of ME. The failure of Cul-3 knockdown to replicate the pan-neuronal seizure effect of inc knockdown or knockout suggests that *inc* has a non CRL-3 function underlying this effect. Further work to establish the molecular interactors and specific neuronal physiology affected by *inc* will shed light on this exciting finding.

### 4.3. Overexpression of Inc

To assess whether the inc effects on seizures were fully malleable by inc levels, we used UAS-inc to overexpress the protein in an *ATP6^1^* animals. While it may be unlikely that overexpression of one component of a multi-protein complex would have an effect without the concurrent upregulation of its binding partners, if inc binding to its substrates is a rate-limiting step, the activity of the entire complex could be sped up by increased availability. It is also worth noting that Cul3 did not mimic the inc effect on seizures, and thus a non-Cullin RING Ligase function of inc is a mechanistic possibility.

Overexpression of inc in cholinergic neurons had the same effect as RNAi knockdown in the same population—exacerbation of seizures compared to the age-matched driver controls, although the effect is qualitatively milder and not significant at Day 18. This shared worsening effect may reflect that imbalance in an inc-mediated process, whether due to too much or too little, ultimately results in similar changes on a downstream cellular process, or that inc may modulate different processes that ultimately produce the same emergent behavior.

Overexpression of inc in dopaminergic neurons resulted in a qualitative trend toward seizure improvement, but this effect did not reach significance at any tested time point. In contrast, in GABAergic neurons, the overexpression of inc resulted in the opposite phenotype as the knockdown, with exacerbated seizures but maintenance of the KD response in advanced disease. It may be a reasonable hypothesis that if reduced expression of *inc* induces synaptic overgrowth, overexpression may reduce the number of GABAergic synapses, lowering inhibitory tone. Alternatively, a UPS-mediated process may be increasing vs. decreasing enzymes necessary for GABA synthesis or synaptic release depending upon available inc. Further study will be needed to discern the underlying mechanism, but this result suggests GABAergic signaling is dynamically regulated by an inc-mediated process and, more broadly, may suggest GABAergic neurons are a target of interest for determining mitochondrial encephalomyopathy seizure etiology and effective therapy.

### 4.4. Mechanistic Considerations for Inc Modulation of ATP6^1^ Seizures

The *Cul3* knockdown results indicate that the inc and Cul3 effects on baseline seizure severity and response to the KD are separable, and could suggest that inc is operating via two distinct mechanisms, one that is dependent on Cul3 (KD mediation), and one that is mechanistically distinct (seizure severity). However, this should be interpreted with caution as pan-neuronal knockdown of a critical cellular homeostasis adaptor could have distinct, complex, or even conflicting effects on overall excitability within neuronal subpopulations. However, it should be noted that the mammalian orthologs of *inc* are known to have non-UPS effects [86]. KCTD5, for example, sequesters beta gamma subunits of GPCRs, regulating their downstream signaling ability, especially with cAMP [30,87,88]. It also interacts with the zip14 zinc transporter, modulating influx of zinc and consequently affecting adenylyl cyclase function [89,90]. KCTD17 interacts with the cytoskeleton in a non-UPS mechanism that can promote projection growth [91], and other KCTD family members can alter potassium channel kinetics in the hippocampus [92].

Much mechanistic light could be shed on inc’s role in seizures by a more thorough understanding of its binding partners, whether they are targets for degradation, sequestration, or mono-ubiquitination, which is an important function of inc at the *Drosophila* neuromuscular junction [44] and can regulate broad cellular homeostatic processes like DNA repair, gene transcription, trafficking, and signal transduction [1,93,94]. While a complete list remains to be determined, some interacting proteins of inc and Cul3 are known. For example, the inc/Cul3 complex likely interacts with co-adaptor protein Peflin to affect retrograde signaling at the NMJ in a Ca-dependent manner [44]. Other interacting genes include *cheerio*, which impacts neuronal growth cones, *Myo10A*, which expresses an unconventional myosin in neurons and glia, and *Grasp65*, encoding a Golgi organization protein that may be involved in dendrite mophogenesis [44,95]. Cul3 may interact with the HCN2 ion channel in the mammalian ventral tegmental area [33], and, while KCTD13 is not an inc ortholog, it has been shown to regulate seizure activity by targeting the GluN1 subunit of the NMDA receptor for proteasomal degradation [96], suggesting a highly plausible mechanism for modulation of excitability by inc.

Also worthy of mechanistic consideration is the relationship between the UPS and mitochondria, given that *ATP6^1^* is a mitochondrial encephalomyopathy mutant. As noted above, mitochondria are subjected to proteasomal control in several ways, including being targeted for mitophagy. Since heteroplasmy in *ATP6^1^* is extremely high [47,78] and likely results in a non-functional version of Complex V, it is unlikely that breaking down fewer of the organelles would be helpful for energy production; however, other mitochondrial functions are important for neuronal health, including regulation of calcium dynamics and apoptosis. Additionally, we have shown that the citric acid cycle, which takes place in the mitochondrial matrix, is critically important for regulating the neurological function of *ATP6^1^*, and that its upregulation is a therapeutic benefit of the ketogenic diet [51,52]. Testing the effect of *inc* loss in other metabolic and excitability models will help elucidate the importance of direct effects on mitochondria in seizure and dietary therapy.

### 4.5. Ketogenic Diet Seizure Reduction Reliance on Functional Inc/Cul3

The ketogenic diet has been used clinically to treat refractory epilepsy for many years, but it is increasingly recognized to positively impact an impressive list of neurological, neurodegenerative, and metabolic conditions, and can even improve health parameters in non-diseased individuals [59,64]. Because the ketogenic diet induces a vast array of downstream effects on metabolism, gene transcription, enzyme and channel regulation, mitochondrial homeostasis, inflammation, oxidative state, and more, it is difficult to know exactly which effects are responsible for its therapeutic effects in each disease state. Because of this, we undertook an RNAi screen of channels, receptors, and other small molecules known to play roles in excitability to look for modifiers of the ketogenic diet response in *ATP6^1^*. *Inc* emerged as a positive genetic interactor, which we validated with the classical allele *inc^1^*.

In contrast to the basal effect on seizures, the failure of the ketogenic diet in pan-neuronal or global inc knockout flies is replicated in the pan-neuronal knockdown of *Cul3*, suggesting that this mechanism is likely to involve Cullin RING Ligase-mediated protein degradation. Additionally, although the effects of different neuronal subtypes on seizures varied with location and neurotransmitter, all tested subpopulation-restricted knockdowns of *inc* resulted in an ablated ketogenic diet response. This suggests that seizure amelioration requires widespread cooperation among neuronal types, and that perturbation in one circuit may be enough to break down the therapeutic effect. However, another possibility that must be considered is that, in cases where *inc* loss reduces seizure phenotype (GABAergic and dopaminergic neurons), the ketogenic diet runs into a “ceiling effect”, beyond which no further reductions to seizures are possible due to the underlying pathology. Indeed, several cases can be noted in the data where *inc* knockdown on regular media brings the TTR to approximately the same level as the ketogenic diet brings the controls (DA Days 18 and 21: GABAergic Days 15 and 18) (Figure 3C,D). This stands in contrast to *inc* knockdown in the mushroom bodies or generally in cholinergic neurons, where worsened seizure phenotypes had plenty of room for improvement, but the KD could not accomplish this in the face of knocked down *inc*.

If the success of the ketogenic diet is predicated upon the correct functioning of the UPS, and Cul3/inc specifically, it is reasonable to hypothesize that plausible targets may include metabolic enzymes. Those that participate in fatty acid oxidation and other lipid metabolism, anaplerosis, and ketogenesis are all presumably utilized significantly more robustly when fed high-fat ketogenic media rather than a standard carbohydrate-based media, and thus may need to be turned over more frequently. This could also be true for protein targets of ketone body, lipids, and small metabolites, such as ion channels and receptors that mediate excitation and inhibition.

The ketogenic diet. the UPS, and mitochondrial disease may also intersect in the critically important AGE/RAGE cellular pathways. AGEs (advanced glycation end products) are formed when reactive carbonyl compounds such as methylglyoxal (MGO) covalently interact with amino acids and nucleotides, irreversibly damaging them. The primary source of these reactive carbonyls are simple sugars which perform non-enzymatic glycosylation and then undergo molecular transformations while bound to their targets; therefore, an excess reliance on glycolysis, as occurs in *ATP6^1^*, can exacerbate AGE production [97,98]. AGEs are damaging as they are often proteasome resistant, prone to aggregation, and damaging to tissue, including blood vessels and endothelial cells, but they are also the ligands to a class of receptors called RAGE (receptors of AGEs) whose activation unleashes a series of harmful pro-inflammatory and ROS-generating cellular responses, which often catalyzes the production of more AGEs [71,99,100,101]. These modified molecules are known to play roles in diabetes, cardiovascular disease, cancer, and neurodegeneration, as well as accelerating the aging process [67,69,71,72,73,102,103]. In the mitochondria specifically, AGEs can lead to damaged ETC components and mtDNA, increasing proteotoxic and oxidative stress in an already-diseased organelle [104,105,106]. Because AGEs lead to the production of damaged proteins, this pathway interacts critically with the UPS, which is responsible for their removal. This process is often hindered by AGEs’ resistance to normal degradation processes and AGE modifications of proteasomal components [69,107,108]. The KD can ameliorate the production of AGEs by lowering the availability of sugar and reducing glycolytic byproducts such as MGO, reducing carbonyl stress. It also activates the anti-inflammatory Nrf2 gene [62], which boosts the levels of Glo1 and Glo2, enzymes that break down MGO. Therefore, it is a reasonable hypothesis that the KD may be improving *ATP6^1^* phenotypes via reduced AGE and RAGE activation, reducing cellular and tissue damage in ME. However, in the absence of *inc* or *Cul3*, this benefit may be hindered by impairment of the degradation of targets that have been pathologically modified.

The ketogenic diet is also known to affect protein availability and activity at both the transcriptional and post-translational levels; both mechanisms may intersect heavily with proteostasis maintenance by the UPS. For example, the KD can change gene transcription via epigenetic mechanisms, including globally reduced DNA methylation [109], ketone body modulation of histones, either by inhibiting the function of histone deacetylases (HDACs), or modifying them directly by β-hydroxybutyrylation (Kbhb), a recently recognized post-translational modification whereby b-hydroxybutyrate is covalently attached to lysine residues [110,111,112]. Genes upregulated by these modifications include those for lipid metabolism and antioxidation [113,114]. Besides its effects on histones, this modification acts upon metabolic enzymes, including those used for lipid anabolism, glyoxylate production, the Krebs cycle, and oxidative phosphorylation (OXPHOS) [115]. The KD can also modify cellular oxidative stress levels, leading to changes in gene transcription [116]. Indeed, Cul3 is known to target Nrf2, a transcription factor activator of the antioxidant stress response that is activated by the ketogenic diet [62,117]. Upregulated gene transcription via any of these mechanisms occurring concurrently with reduced or absent UPS activity may result in the loss of proteostasis. Finally, ketone bodies have been shown to regulate the unfolded protein response (UPR), in which ER buildup of misfolded proteins triggers changes in transcription and increased activity of the UPS [112,118]. Given all these roles for ketone bodies in protein production, activity, and quality control, it is reasonable to hypothesize that the benefits of the ketogenic diet may require successful integration with the UPS.

### 4.6. Limitations of the Current Study

Results presented here are due to genetic manipulations and measured at the behavioral level, and thus all mechanistic discussion presented above is plausible given current results and known neurophysiology and metabolism, but thus far represents hypothetical mechanistic explanations. Additionally, the genetic manipulations performed here—RNAi, genetic knockout, and transgenic overexpression—may have variable effects on protein levels, which must be investigated before conclusions can be drawn about protein turnover and UPS activity. Future studies should address developmental concerns, effects on metabolic pathways such as glycolysis and ketogenesis, protein turnover, mitochondrial function, and neurophysiological parameters such as excitability and synaptic activity. Further work on mammalian models can address the potential variability in behavioral outcomes between *Drosophila inc* and its mammalian orthologs.

## 5. Conclusions

Overall, we have shown that, in addition to its established roles in sleep, synaptic transmission, and neurodevelopment, the BTB proteasome adaptor *inc* also modifies excitability and neuronal response to metabolic state. We have found that inc modulates the severity of seizures in a model of mitochondrial encephalomyopathy in a non-Cullin-3-dependent manner, revealing a previously unrecognized mechanism of action for this protein. These seizure effects are neuronal-subtype-specific, with seizures exacerbated by genetic knockdown in the mushroom bodies and other cholinergic neurons, but ameliorated with the same manipulations in GABAergic and dopaminergic neurons. Our studies also reveal a role for inc in the ability of the KD to significantly reduce seizure severity in ME; in contrast to the previous result, this role is mimicked by the genetic knockdown of *Cul-3*, implicating inc’s previously established role in mediating proteasomal degradation. These findings establish a novel genetic link between neuronal excitability, neuronal proteostasis, and metabolic modulation by diet and disease. Future investigations will be required to establish the molecular mechanism underlying this genetic link, including identifying specific targets of inc and validating these findings with mammalian orthologs. Further work will also be needed to establish the broader neurological impact of *inc*, including testing its effects on other neurological phenotypes of *ATP6^1^*, including sleep and circadian rhythm defects, and investigating its ability to modulate seizures and dietary response in other disease models.

## Figures and Tables

**Figure 1:**
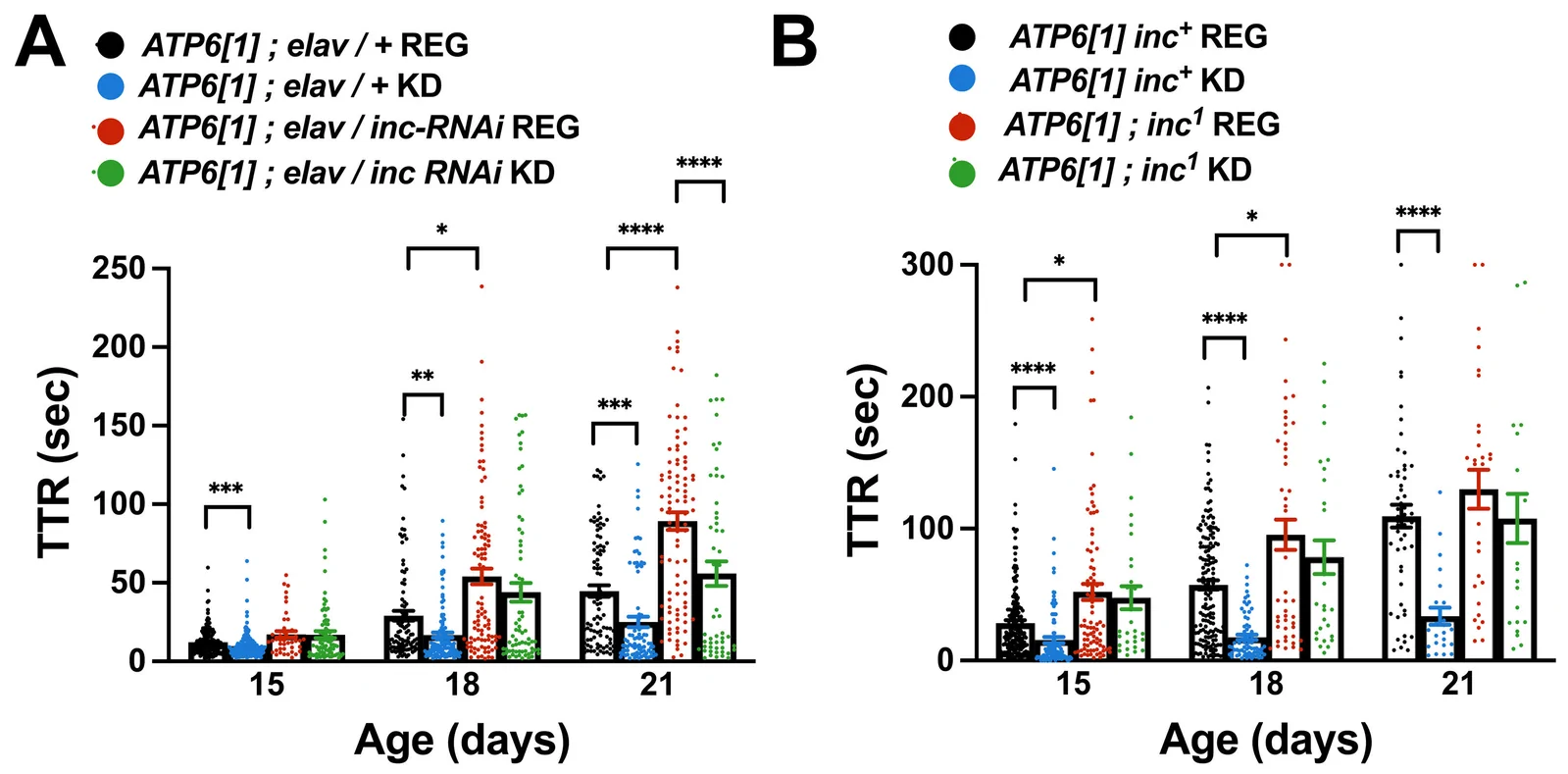
Pan-neuronal knockdown and global genetic knockout of inc in *ATP6^1^* worsens seizures and counteracts the ketogenic diet. (**A**) Pan-neuronal knockout of inc (*ATP6[1]*;; *elavGAL4*/*UAS-RNAi-inc*, red symbols) worsens seizures compared to age-matched controls (*ATP6[1]*;; *elavGAL4*/+, black symbols) on regular diet (RD). At Day 15: 12.3 ± 0.6 (*n* = 167, *p* = 0.2946) vs. 16.8 ± 1.7 (*n* = 79); Day 18: 29.1 ± 3.1 (*n* = 101, *p* = 0.0171) vs. 53.5 ± 5.1 (*n* = 99); Day 21: 44.8 ± 3.7 (*n* = 94 *p* < 0.0001) vs. 89.2 ± 5.7 (*n* = 97). The ketogenic diet (KD) reduces seizures significantly for control genotype at all time points (black vs. blue symbols) but not for inc knockdown flies, red vs. green symbols; Day 15: 17.1 ± 2.1 (*n* = 82, *p* = 0.4336); Day 18: 44.0 ± 5.9 (*n* = 72, *p* = 0.1068); Day 21: 55.8 ± 7.4 (*n* = 53, *p* < 0.0001). (**B**) Global knockout by classical allele (*ATP6[1]*; *inc*^+^ or *inc^1^*) worsens seizures on RD (black vs. red symbols) at Day 15 (28.5 ± 2.1, *n* = 181 vs. 52.1 ± 6.2, *n*= 87) and Day 18 (57.4 ± 3.5, *n* = 162 vs. 99.4 ± 12.2, *n* = 51). The ketogenic diet is robustly effective at reducing seizures at all time points for *ATP6[1]* alone (*p* < 0.0001 at all time points, black vs. blue symbols) but its efficacy is eliminated for *ATP6[1]*; *inc1* double mutants (green symbols; Day 15: 47.5 ± 8.9, *n* = 30, *p* > 0.9999; Day 18: 78.4 ± 13, *n* = 29, *p* > 0.9999; Day 21: 107.7 ± 18.9, *n* = 24, *p* = 0.8676). * is *p* < 0.05; ** is *p* < 0.01, *** is *p* < 0.001, **** is *p* < 0.0001.

**Figure 2:**
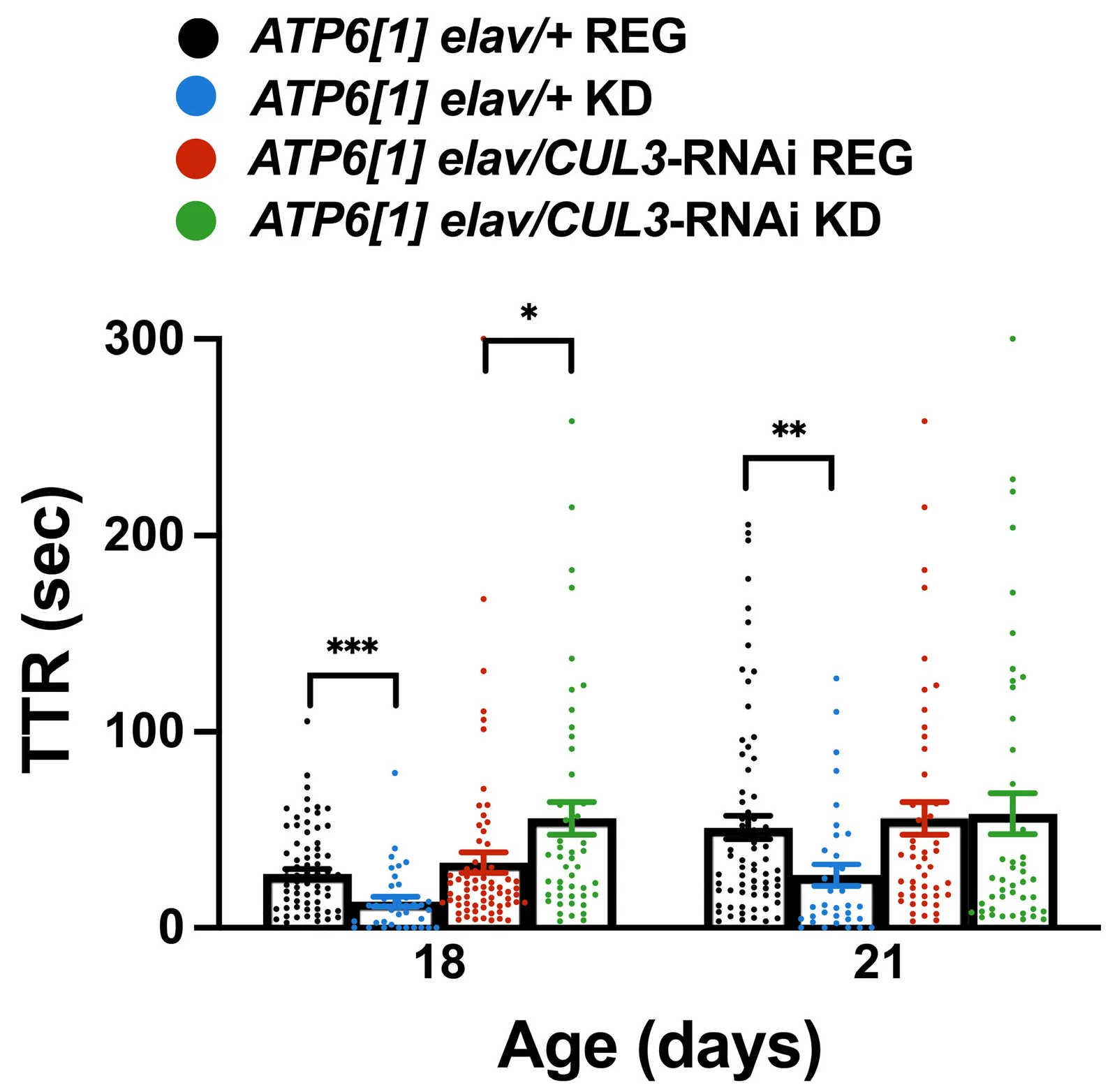
Pan-neuronal knockdown of Cullin-3 does not affect basal seizure severity but prevents KD benefit. At all time points, the KD is effective in reducing seizures significantly in driver-only control flies (*ATP6[1]*;; *elavGAL4*/+, black symbols vs. blue symbols). Day 18: 27.3 ± 2.6 (*n* = 76, *p* = 0.0008) vs. 13.3 ± 2.6 (*n* = 38); Day 21: 51.1 ± 5.9 (*n* = 77, *p* > 0.0001) vs. 27.0 ± 5.6 (*n* = 35). When Cul3 is knocked down (*ATP6[1]*;; *elavGAL4*/*UAS-RNAi-Cul3*), this benefit is reversed or ablated (red vs. green symbols). Day 18: 55.9 ± 8.3 (*n* = 49, *p* = 0.0196); Day 21: 58.2 ± 10.6 (*n* = 47, *p* > 0.9999). * is *p* < 0.05; ** is *p* < 0.01, *** is *p* < 0.001. *Cul3* knockdown, unlike *inc* knockdown, does not worsen seizures compared to driver controls on regular media. Day 18: 33.3 ± 5.5 (*n* = 69, *p* > 0.9999); Day 21: 55.9 ± 8.3 (*n* = 49, *p* > 0.9999).

**Figure 3:**
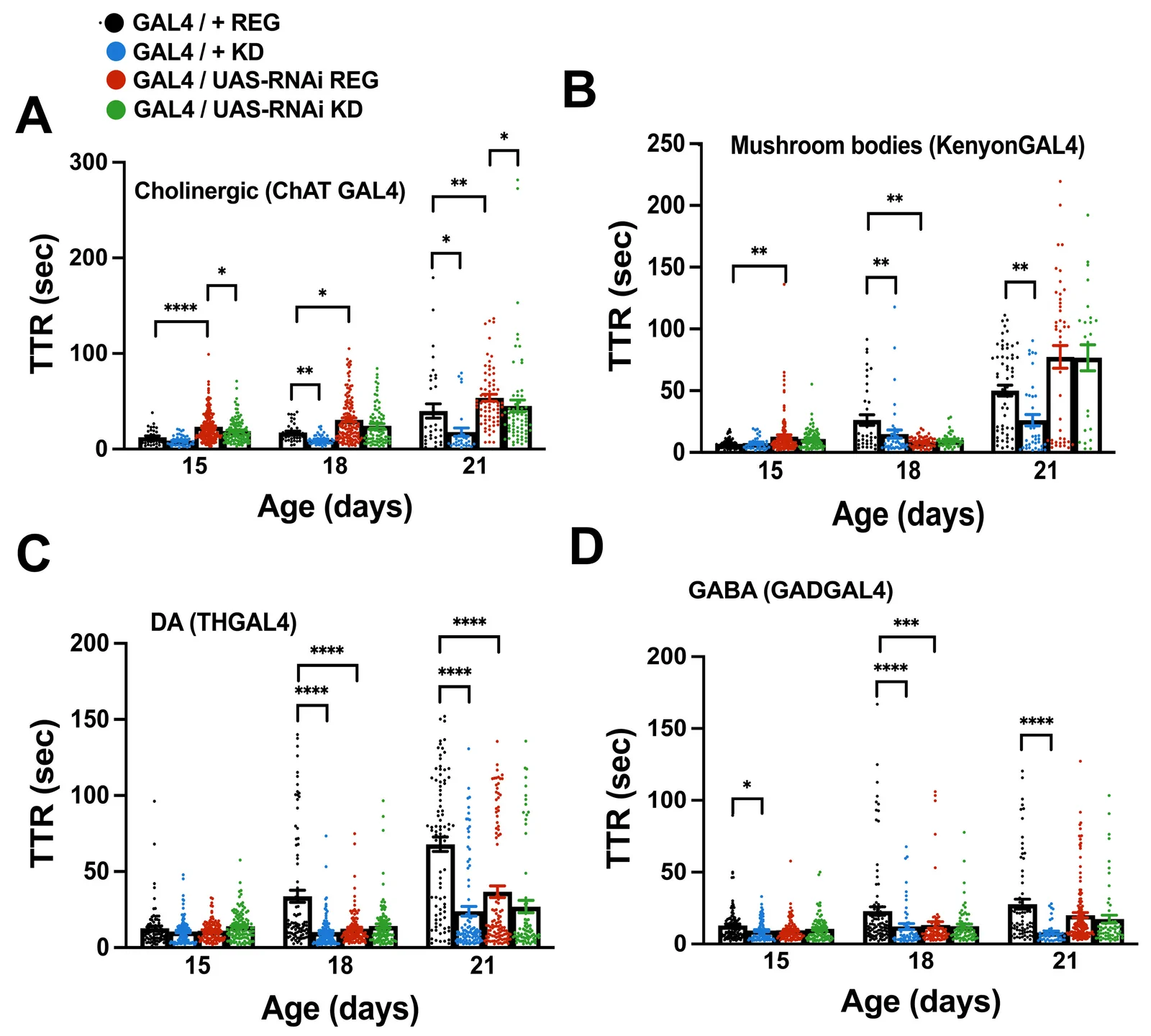
Inc knockdown in neurons has subpopulation-specific effects on seizure severity and response to the ketogenic diet. In all panels, black symbols represent driver-only controls on RD, blue symbols represent driver-only controls on KD, red symbols represent inc knockdown on RD, and green symbols represent inc knockdown on KD. Driver used in each panel is indicated in the figure ((**A**): cholinergic; (**B**): mushroom bodies; (**C**): dopaminergic; (**D**): GABAergic). (**A**) Cholinergic driver control flies (*ATP6^1^*;; *ChATGAL4*/+) on regular media had TTR values of Day 15: 12.5 ± 1.5 (*n* = 33), Day 18: 17.5 ± 1.5 (*n* = 39), and Day 21: 40.0 ± 7.7 (*n* = 34). The KD improved these TTRs to Day 15: 9.1 ± 0.8 (*n* = 42, *p* = 0.4032), Day 18: 9.8 ± 0.8 (*n* = 42, *p* = 0.0037), and Day 21: 17.8 ± 4.5 (*n* = 29, *p* = 0.0161). Inc knockdown (*ATP6^1^*;; *ChATGAL4*/*UAS-RNAi-inc*) worsened seizures on RD compared to RD controls, Day 15: 23.3 ± 1.1 (*n* = 193, *p* < 0.0001), Day 18: 30.6 ± 2.0 (*n* = 140, 0.0292), and Day 21: 53.7 ± 3.6 (*n* = 72, *p* = 0.0017). In this genetic background, the KD was less effective, Day 15: 19.0 ± 1.1 (*n* = 122, *p* = 0.0458), 24.4 ± 2.2 (*n* = 83, *p* = 0.0796), 45.1 ± 6.2 (*n* = 68, *p* = 0.0108). (**B**) Mushroom body driver control flies (*ATP6^1^*;; *KenyonGAL4*/+) on regular media had TTR values of Day 15: 7.0 ± 0.5 (*n* = 57), 26.2 ± 4.4 (*n* = 38), 50.1 ± 4.4 (*n* = 62). The KD improved these TTRs to Day 15: 7.6 ± 0.9 (*n* = 32, *p* = 0.9999), Day 18: 14.8 ± 3.4 (*n* = 44, *p* = 0.0087), and Day 21: 26.1 ± 4.6 (*n* = 38, *p* = 0.0061). Inc knockdown (*ATP6^1^*;; *KenyonGAL4*/*UAS-RNAi-inc*) had complex effects in the mushroom bodies, worsening seizures early Day 15: 12.8 ± 1.4 (*n* = 133, *p* = 0.0061), improving seizures in intermediate disease 8.5 ± 0.6 (*n* = 55, *p* = 0.0019), and trending toward worsening them again at Day 21: 72.4 ± 9.3 (*n* = 45), though this effect does not reach significance (*p* = 0.1178). The KD had no effect on MB knockdown flies, Day 15: 10.9 ± 1.0 (*n* = 74), Day 18: 9.9 ± 0.9 (*n* = 44), Day 21: 76.7 ± 10.7 (*n* = 26), *p* > 0.9999 at all time points. (**C**) Dopaminergic driver control flies (*ATP6^1^*;; *THGAL4*/+) on regular media recovery times were Day 15: 12.8 ± 1.4 (*n* = 87), Day 18: 33.7 ± 4.0 (*n* = 91), Day 21: 68.0 ± 4.8 (*n* = 88). The KD was highly effective on Days 18 and 21. Day 15: 10.6 ± 0.9 (*n* = 102, *p* = 0.5380), 10.2 ± 1.0 (*n* = 102, *p* < 0.0001), 23.9 ± 3.1 (*n* = 95, *p* < 0.0001). Inc knockdown in dopaminergic neurons (*ATP6^1^*;; *THGAL4*/*UAS-RNAi-inc*) reduced seizure severity at intermediate and late time points compared to driver controls, Day 15: 11.1 ± 0.6 (*n* = 113, *p* > 0.9999), 12.4 ± 1.0 (*n* = 118, *p* < 0.0001), 36.7 ± 3.9 (*n* = 110, *p* < 0.0001) but eliminated the KD response at all time points, Day 15: 14.1 ± 0.9 (*n* = 116, *p* = 0.1434), 14.3 ± 1.4 (*n* = 113, *p* > 0.9999), 27.0 ± 4.1 (*n* = 76, *p* = 0.1572). (**D**) GABAergic control flies (*ATP6^1^*;; *GADGAL4*/+) on regular media recovery times were Day 15: 12.9 ± 1.2 (*n* = 109), Day 18: 23.0 ± 3 (*n* = 102), and Day 21: 27.7 ± 3.5 (*n* = 75). The KD improved these TTRs at all time points, Day 15: 9.4 ± 0.75 (*n* = 90, *p* = 0.0171), 12.2 ± 2.0 (*n* = 56, *p* < 0.0001), 8.1 ± 1.1 (*n* = 43, *p* < 0.0001). Knockdown of *inc* in these neurons (*ATP6^1^*;; *GADGAL4*/*UAS-RNAi-inc*) trended TTRs downward at all time points, but reached significance only at Day 18—Day 15: 9.6 ± 0.8 (*n* = 98, *p* = 0.1407), 13.3 ± 2.3 (*n* = 79, *p* = 0.0005), 20.1 ± 1.8 (*n* = 152, *p* = 0.3925). The KD amelioration is absent in these knockdowns, Day 15: 10.6 ± 0.9 (*n* = 87, *p* > 0.9999), 12.4 ± 1.5 (*n* = 72, *p* > 0.9999), 17.3 ± 2.8 (*n* = 62, *p* = 0.1584). * is *p* < 0.05; ** is *p* < 0.01, *** is *p* < 0.001, **** is *p* < 0.0001.

**Figure 4:**
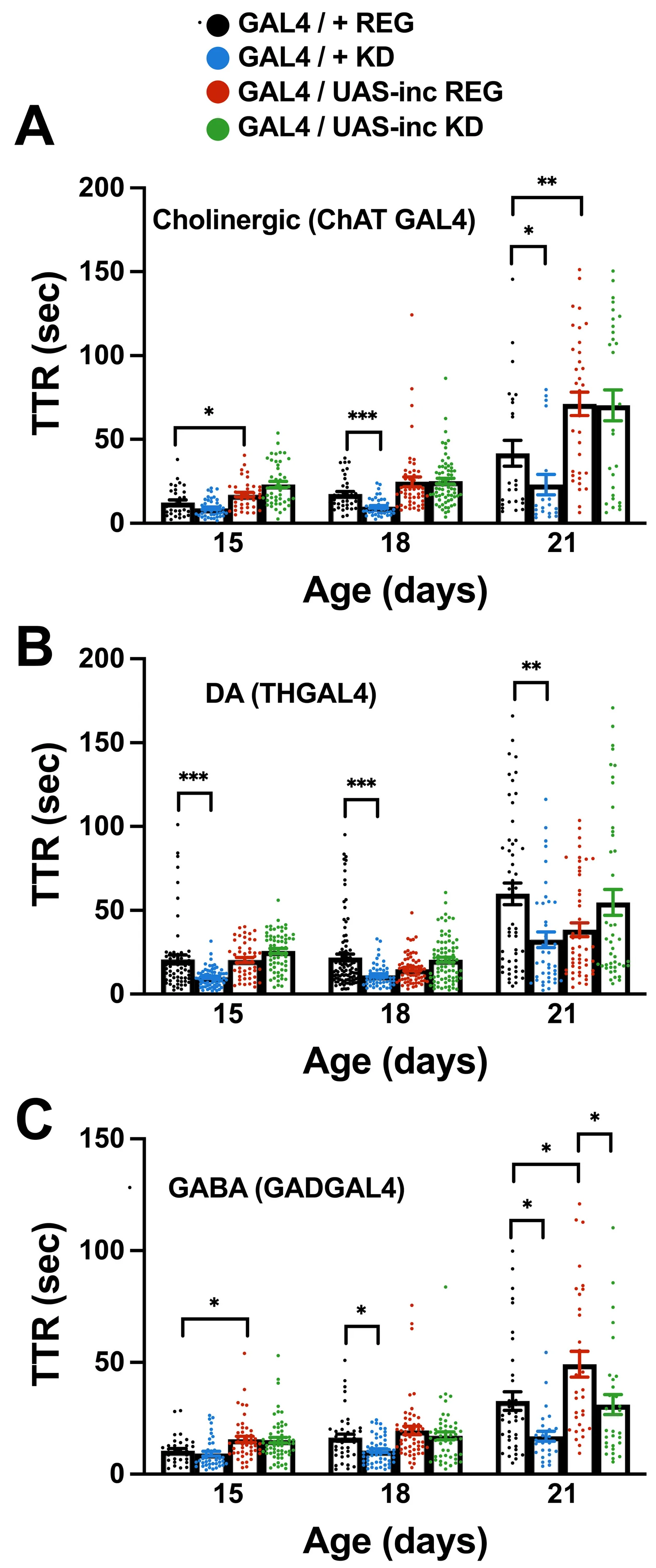
Overexpression of inc in cholinergic, dopaminergic, and GABAergic neurons modifies seizure phenotypes. All panels are color-coded as follows: Driver controls RD—black symbols; driver controls RD—blue symbols; overexpressers RD—red symbols; overexpressers KD—green symbols. (**A**) Overexpression of inc in cholinergic neurons (*ATP6^1^*;*inc*^+^; *ChATGAL4*/*UASi-inc*) worsened seizures at Days 15 and 21 compared to driver controls. Overexpressers, Day 15: 17.3 ± 1.4 (*n* = 37), 24.7 ± 2.9 (*n* = 50), 71.2 ± 7.1 (*n* = 35) vs. controls, Day 15: 12.5 ± 1.5 (*n* = 33, *p* = 0.0436), Day 18: 17.5 ± 1.5 (*n* = 39, *p* = 0.2423), and Day 21: 40.0 ± 7.7 (*n* = 34, *p* = 0.0035). These overexpressing flies lacked a response to the ketogenic diet, Day 15: 23.1 ± 1.9 (*n* = 46, *p* = 0.3710), 25.0 ± 1.7 (*n* = 70, *p* = 0.9317), 70.35 ± 9.3 (*n* = 30, *p* > 0.9999). (**B**) Dopaminergic driver controls, Day 15: 20.8 ± 2.7 (*n* = 62), 21.9 ± 2.0 (*n* = 103), 59.9 ± 6.5 (*n* = 52) responded to the KD as expected, Day 15: 10.0 ± 0.7 (*n* = 69, *p* = 0.0004), 11.2 ± 1.01 (*n* = 49, *p* = 0.0006), 32.4 ± 4.8 (*n* = 40, *p* = 0.0045). Overexpression of inc in dopaminergic neurons (*ATP6^1^*;*inc*^+^; *THGAL4*/*UAS-inc*) did not change seizure severity compared to age-matched controls, Day 15: 20.4 ± 1.5 (*n* = 49, *p* = 0.3299), 15.0 ± 1.0 (*n* = 67, *p* = 0.5847), 38.4 ± 4.2 (*n* = 56, *p* = 0.1438). These flies did not respond to the KD, Day 15: 25.7 ± 1.5 (n = 64, *p* = 0.1397), 20.5 ± 1.5 (*n* = 81, *p* = 0.1057), 54.7 ± 2.8 (*n* = 44, *p* = 0.8094). (**C**) GABAergic driver controls, Day 15: 10.5 ± 1.0 (*n* = 35), 16.3 ± 1.7 (n = 43), 32.7 ± 4.2 (*n* = 38) responded to the KD at Days 18 and 21, Day 15: 9.0 ± 1.0 (*n* = 49, *p* = 0.4625), 10.9 ± 0.9 (*n* = 46, 0.0388), 17.1 ± 2.3 (*n* = 26, *p* = 0.0198). Overexpression of inc in GABAergic neurons (*ATP6^1^*;*inc*^+^; *GADGAL4*/*UAS-inc*) worsened seizures at early and late time points, Day 15: 15.6 ± 1.4 (*n* = 47, *p* = 0.0212), 19.6 ± 1.9 (*n* = 55, *p*= 0.4823), 49.2 ± 5.9 (*n* = 32, *p* = 0.0486). These flies responded to the KD at Day 21 only, Day 15: 15.2 ± 1.3 (*n* = 59, *p* > 0.9999), 17.1 ± 1.8 (*n* = 51, *p* = 0.8396), 31.1 ± 4.5 (*n* = 32, *p* = 0.0313). * is *p* < 0.05; ** is *p* < 0.01, *** is *p* < 0.001.

**Table 1:** Summary of inc neuronal subpopulation RNAi knockdown and transgenic overexpression results. Basal seizure effects are designated “worse” (red) when seizure TTR is significantly higher at at least one tested time point and “reduced” (green) when TTR is significantly lower at at least one time point. For KD efficiency, cells are designated “ablated” (red) when no significant reduction in TTR by KD treatment occurred at any time point and “less” (light red) when this was true at one or two time points, but not all. “No effect” (yellow) signifies no significance in TTR at any timepoint for that condition.

	Knockdown (*inc RNAi*)		Overexpression (*UAS-inc*)	
Population	Seizure Severity	KD Efficacy	Seizure Severity	KD Efficacy
Pan-neuronal	worse	less	not tested	not tested
GABAergic	reduced	ablated	worse	less
Dopaminergic	reduced	ablated	no effect	ablated
Cholinergic	worse	less	worse	ablated
M. Bodies	worse	ablated	not tested	not tested

## Data Availability

All data being reported are complete data sets. Additional information and protocols are available upon request.

## Abbreviations

The following abbreviations are used in this manuscript:

KD	Ketogenic Diet
RD	Regular Diet
inc	Insomniac
Cul-3	Cullin-3
